# Detecting N-Phenyl-2-Naphthylamine, L-Arabinose, D-Mannose, L-Phenylalanine, L-Methionine, and D-Trehalose via Photocurrent Measurement

**DOI:** 10.3390/gels10120808

**Published:** 2024-12-09

**Authors:** Feng Li, Ruoxi Yang, Jian Xu, Guohai Xu, Ye Wu

**Affiliations:** 1 School of Electrical and Automation Engineering, Nanjing Normal University, Nanjing 210046, China; 2 Division of Electrical and Computer Engineering, Louisiana State University, Baton Rouge, LA 70803, USA; 3 Key Laboratory of Jiangxi University for Functional Materials Chemistry, School of Chemistry and Chemical Engineering, Gannan Normal University, Ganzhou 341000, China

**Keywords:** small molecule detection, photocurrent, point-of-care, sensitivity

## Abstract

The concentration of small molecules reflects the normality of physiological processes in the human body, making the development of simple and efficient detection equipment essential. In this work, inspired by a facile strategy in point-of-care detection, two devices were fabricated to detect small molecules via photocurrent measurement. A linear response of the photocurrent against the concentration of the small molecules was found. In the first device, metal ions were introduced into gel substrates made by xanthan gum to enhance photocurrent response. N-phenyl-2-naphthylamine was measured when iron or manganese ions were used. L-Phenylalanine was measured when the gel was modified by samarium, iron, cerium, or ytterbium ions. L-(+)-Arabinose was detected via the gels modified by iron or holmium ions. D-(+)-Mannose was detected when the gel was modified by ytterbium, manganese, chromium, or sodium ions. L-Methionine was detected in the gels modified by samarium, zinc, or chromium ions. The second device was based on a paper sheet. A sugar-like molecule was first synthesized, which was then used to modify the paper. The detection was possible since the photocurrent showed a linear trend against the concentration of D-Trehalose. A linear fit was conducted to derive the sensitivity, whose value was found to be 5542.4. This work offers a novel, simple, and environmentally sustainable platform that is potentially useful in remote areas lacking medical devices.

## 1. Introduction

The high standards of modern life put forward a variety of requirements for the detection of small molecules, which is related to advanced medical care, food safety control, and precision treatment. Small molecules are an important part of living organisms and participate in important physiological processes in the human body. Their concentration is closely related to the diagnosis, prognosis, and therapeutic effect of many diseases [1,2,3]. This can be seen in multiple biomarker tests, where cancer diagnosis is required. Clinicians can use the level of the small molecules to obtain enough information and make an accurate diagnosis. A good example can be found in DNA and RNA mutation detection, where the detection of relatively small molecules such as reactive oxygen species and reactive nitrogen species can provide auxiliary functions for diagnosis [4]. Indeed, the level of the small molecules is related to the occurrence of the diseases. One example can be seen in detecting the levels of blood glucose and ketone, which can reflect the progression of diabetes. Another example can be found in elevated uric acid levels that are closely associated with serious diseases such as gout. Similarly, microRNA tests can be used to rapidly identify and monitor infectious agents.

Typical methods for detecting the small molecules include gas chromatography, mass spectrometry, high-performance liquid chromatography [5,6,7,8], UV-visible spectrophotometry [9,10], and capillary electrophoresis [11,12], among others. However, these methods require expensive instruments. Moreover, they are complex to operate and time-consuming. This means that their applicability is limited. It should be noted that people in remote areas have limited access to advanced analytical or medical equipment, which makes these methods fail to meet practical needs.

Point-of-care (POC) testing is a good idea for small molecule detection. POC testing refers to the method of real-time detection by portable analytical instruments and reagents at the sampling site, which has the advantages of simple measuring instruments, controllable cost, fast detection, and is maintenance free. Some teams have applied POC strategies to design simple and effective devices for the detection of the small molecules. Ramses V. Martinez et al. designed a self-powered, paper-based electrochemical device that enables the electrochemical detection of glucose, uric acid, and L-lactic acid in real time and with accuracy. The top layer of their device was made of cellulose paper with a patterned hydrophobic domain for a self-pipetting test area for electrochemical detection. The bottom layer of their device is a friction generator manufactured on hydrophobic paper that is capable of collecting electrical energy from the user’s interaction. The device is inexpensive, easy to use, and self-powered [13]. Lee, J.R. et al. designed a device for detecting tetrahydrocannabinol (THC) in saliva that uses a giant magnetoresistance biosensor integrated with a portable reading system and cellphone. It can be used to measure THC in the range of 0 to 50 ng/mL through a simple saliva collection scheme. This work facilitates the immediate detection of THC and shows the potential of applying POC strategies to test other small molecules [14].

Some groups have used commercial instruments to construct biochemical testing platforms. Zhong, Q. et al. used a POC fluorescent reader for detecting early-state tumors. Their method did not require high costs of medical imaging facilities and reduced resource disparities for the early detection of lung cancer [15]. Banbury, C. et al. utilized a Raman spectrometer combined with an artificial neural network algorithm to acquire molecular fingerprints of traumatic brain injury [16].

Their work is inspiring. From this, the following question may arise. Can we make a device that is similar to these POC devices with the characteristics of simple fabrication and high selectivity?

In the general detection of small molecules, the sensitivity of the target materials is very important. This sensitivity may be related to conductivity, given that we may prefer to use the method of the photocurrent. The general materials used for introducing the conductivity may fall into the category of carbon nanomaterials, including carbon dot, carbon nanotubes, graphite, and graphene. These materials hold excellent optical properties, which have been widely used to amplify fluorescence polarization. The high sensitivity of detecting small molecules can be associated with the anisotropy signals of the fluorescence polarization for these materials. However, these materials can hold limited biosafety due to their inherent inorganic properties.

Gel materials have been regarded as types of small-molecule detection materials with good performance due to their characteristics of high sensitivity, good biocompatibility, fast response, and environmental friendliness [17,18,19]. Since the gel itself is a material with complex molecular bonds and groups, their presence creates opportunities for the introduction of defects. These advantages make the gel an ideal material for the generation of a photocurrent. In our experience, xanthan gum can be useful as a matrix for introducing a solution of small molecules. The xanthan gum is a well-known material with biocompatibility. These advantages let us choose xanthan gum as the substrate. The gel materials can be photoresponsive [20,21]. Under light irradiation, electronic charge pairs will be formed on the surface of the gel due to defects. Negative charges and positive charges gather separately to form their own regions. These regions form the electric polarization parameters, respectively, which are directly proportional to the current. Thus, multiple photocurrents can be generated. The superposition of these photocurrents forms a total photocurrent that can be measured by the instrument. The introduction of metal ions into the xanthan gum increases the presence of surface defects. This further introduces an internal electric field, which induces and enhances the generation of the photocurrent. The introduction of different metal ions may lead to various levels of aggravation of the surface defects, which in turn may result in either an enhanced or suppressed photocurrent. At the same time, the target small molecules added dropwise will aggravate the presence of the surface defects, introduce the built-in electric field, and generate the photocurrent. Due to the existence of the small molecules with different concentrations, the introduced photocurrent is different. Therefore, the value of the photocurrent measured can reflect the concentration of the small molecules.

It is noted that N-phenyl-2-naphthylamine can be used as a general antioxidant and stabilizer for various synthetic rubbers during processing and storage [22,23]. D-(+)-Mannose is a monosaccharide that cannot be hydrolyzed in human bodies. Therefore, it can be used as a sugar substitute for avoiding diabetics, as it does not participate in human metabolism. It can be used to prevent recurrent urinary tract infections, inhibit cancer cell growth, treat immune system diseases, and improve intestinal flora balance [24,25]. L-(+)-Arabinose is a pentose that is barely absorbed by the human body. It does not cause blood sugar spikes and does not convert to fat. L-(+)-Arabinose has roles in regulating blood lipids, improving skeletal muscle, and suppressing obesity [26]. L-phenylalanine is one of the essential amino acids in the human body involved in the synthesis of neurotransmitters and hormones, as well as carbohydrate and fat metabolism [27]. L-methionine is one of the essential amino acids constituting the human body involved in protein synthesis and is closely related to the metabolism of various sulfur-containing compounds in organisms [28,29]. Therefore, accurate detection of the concentration of these small molecules is crucial.

Paper can be considered another candidate for making the substrates related to the detection of small molecules given that they are environmentally friendly and flexible [30]. We plan to modify the paper using certain molecules, which can introduce photo-electronic response.

Herein, inspired by the POC detection strategy, we designed two devices for detecting small molecules. The devices mainly consist of a red-light source, a substrate, and an ampere meter. The substrate was made by a gel and a piece of paper. The red-light source system is introduced by combining a red-light filter with a white-light source from a cell phone. Droplets of the small molecules were added to the substrate. Under the irradiation of red light, the photocurrent generated by different concentrations of the small molecules was varied. It is worth mentioning that the components used in the inspection process were low cost and easily available, including a USD 3.80 red-light filter and an ammeter available on Amazon. These advantages make the devices very useful for real-world applications in remote areas. In addition, the main materials used are the gel and the paper, which are sustainable and environmentally friendly.

The rest of this article is arranged as follows: Section 2 shows the photocurrent obtained using our device and describes the change of the photocurrent with concentration of the small molecules. Section 2 presents the results of this work. Section 4 is the materials and methods. Finally, we summarize our devices in Section 3.

## 2. Results and Discussion

### 2.1. The Detection of Small Molecules via the Gel Substrates

**Figure 1:**
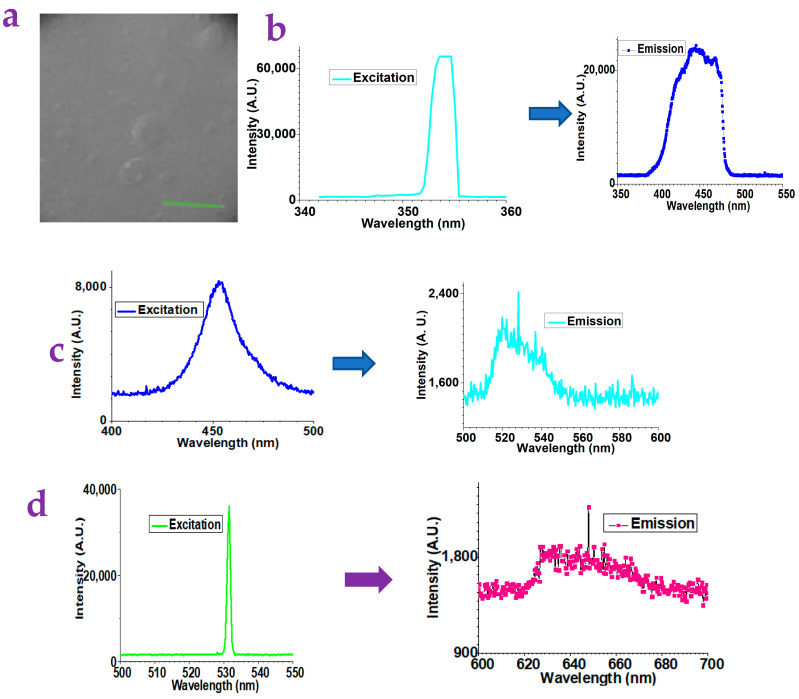
(**a**) Typical surface of xanthan gum substrates as acquired by a lab-built optical microscope (scale bar: 1 mm). (**b**) The xanthan gum substrates without metal ions modification can show strong emission of blue fluorescence when they are excited by a UV light. (**c**) The xanthan gum substrates without metal-ions modification can show weak emission of green light when they are excited by a blue light. (**d**) The xanthan gum substrates without metal-ions modification can show weak emission of red fluorescence when they are excited by a green light.

Figure 1a illustrates the representative surface of the xanthan gum substrates. Figure 1b–d show the fluorescence spectroscopy of the xanthan gum with various excitations. It can be found that these xanthan gum substrates show blue, green, and red emissions when they are excited by the UV light, the blue light, and the green light. It should be mentioned that the blue-light emission is very strong while the green and red emission is weak.

**Figure 2:**
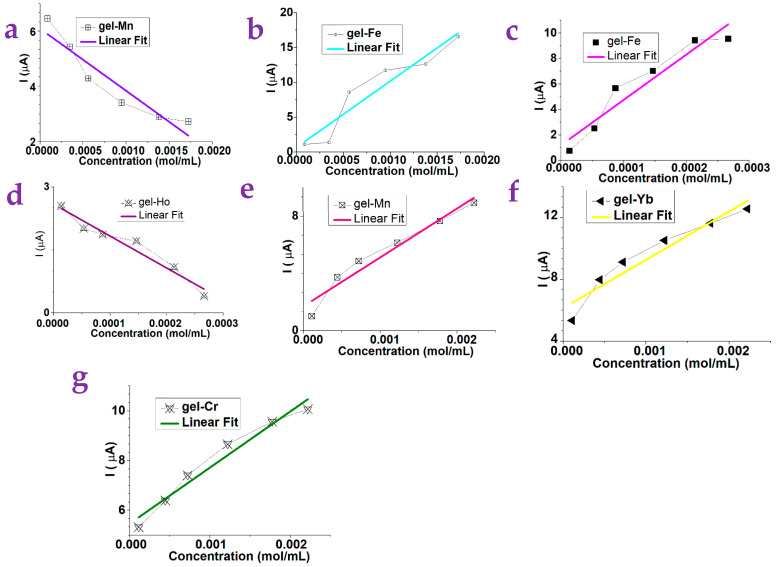
The concentration of the small molecules is found to be dependent on the photocurrent of various gel substrates. (**a**) The photocurrent of the gel-Mn substrate was dependent on the concentration of N-phenyl-2-naphthylamine. (**b**) The photocurrent of the gel-Fe substrate was dependent on the concentration of the N-phenyl-2-naphthylamine. (**c**) The photocurrent of the gel-Fe substrate was dependent on the concentration of L-(+)-Arabinose. (**d**) The photocurrent of the gel-Ho substrate was dependent on the concentration of L-(+)-Arabinose. (**e**) The photocurrent of the gel-Mn substrate was dependent on the concentration of D-Mannose. (**f**) The photocurrent of the gel-Yb substrate was dependent on the concentration of the D-Mannose. (**g**) The photocurrent of the gel-Cr substrate was dependent on the concentration of the D-Mannose. Here, a linear trend was observed.

Figure 2a–g show the test results of the xanthan gum substrates containing different metal ions. It can be seen that the photocurrent of the gel-Mn was suppressed linearly with respect to the increasing concentration of N-phenyl-2-naphthylamine (Figure 2a) while the photocurrent of the gel-Fe was enhanced linearly with respect to the increasing concentration of the N-phenyl-2-naphthylamine (Figure 2b). The sensitivity can be calculated from the absolute value of the slope of the fitting line, which is 2254.3 and 9506.8 for the gel-Mn and the gel-Fe, respectively. Figure 2c,d illustrate the results of L-(+)-Arabinose. The photocurrent of gel-Fe as a test material increased linearly with increasing concentration (Figure 2c), and the photocurrent of gel-Ho as a test material suppressed linearly with increasing concentration (Figure 2d). The sensitivity of the device to detect changes in the concentration of L-(+)-Arabinose solution when gel-Fe and gel-Ho were used can be calculated to be 356,273.5 and 7594.0, respectively.

Figure 2e–g show the test results of the D-Mannose with respect to gel substrates containing different metal ions. Compared with gels containing other metal ions, when the gel-Yb, the gel-Mn, and the gel-Cr are used, the detection results of this device have a better linear fitting degree. Among them, the photocurrent of the gel-Yb, the gel-Mn, and the gel-Cr basically increased linearly with increasing concentration. The sensitivity of the device to detect changes in the concentration of the D-(+)-Mannose when gel-Yb, the gel-Mn, and the gel-Cr were used can be calculated to be 3128.0, 3422.3, and 2252.3, respectively.

**Figure 3:**
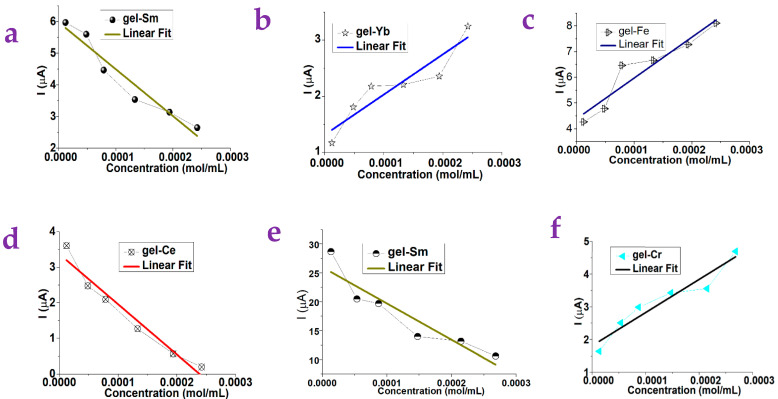
The concentration of the small molecules is found to be dependent on the photocurrent of various gel substrates. (**a**) The photocurrent of the gel-Sm substrate was dependent on the concentration of the L-Phenylalanine. (**b**) The photocurrent of the gel-Yb substrate was dependent on the concentration of the L-Phenylalanine. (**c**) The photocurrent of the gel-Fe substrate was dependent on the concentration of the L-Phenylalanine. (**d**) The photocurrent of the gel-Ce substrate was dependent on the concentration of the L-Phenylalanine. (**e**) The photocurrent of the gel-Sm substrate was dependent on the concentration of the L-Methionine. (**f**) The photocurrent of the gel-Cr substrate was dependent on the concentration of the L-Methionine. Here, a linear trend was observed.

Figure 3a–d show the test results of L-Phenylalanine. The photocurrent of the gel-Fe and the gel-Yb increased linearly with increasing concentration, and the photocurrent of the gel-Ce and the gel-Sm suppressed linearly with increasing concentration. The sensitivity of the device to detect changes in the concentration of L-Phenylalanine solution when gel-Sm, gel-Fe, gel-Ce, and gel-Yb were used can be calculated to be 14,834.5, 15,871.3, 14,126.2, and 7174.5, respectively.

Figure 3e, f show the results of L-Methionine. The photocurrent of gel-Cr increased linearly with increasing concentration, and the photocurrent of gel-Sm was suppressed linearly with increasing concentration. The sensitivity of the device to detect changes in the concentration of L-Methionine solution when the gel-Sm and gel-Cr were used can be calculated to be −62,908.8 and 10,161.3, respectively.

We tested 18 kinds of gels substrates, including gel-Cr, gel-Na, gel-Eu, gel-Y, gel-Sm, gel-Cu, gel-Ce, gel-Ca, gel-Zn, gel-Fe, gel-Ho, gel-Nd, gel-Li, gel-Er, gel-Cs, gel-Yb, gel-Mn, and gel-K. A total of five kinds of small molecules were used as target molecules, including the N-Phenyl-2-naphthylamine, the L-Arabinose, the D-Mannose, the L-Phenylalanine, and the L-Methionine. Table 1, Table 2, Table 3, Table 4 and Table 5 summarize the dependence on the concentration of these small molecules with respect to the photocurrent of gels. The typical linear dependence is plotted on Figure 2 and Figure 3 while the nonlinear dependence is shown in Figures S1–S5.

### 2.2. The Detection of Small Molecules via the Paper Substrates

**Figure 4:**
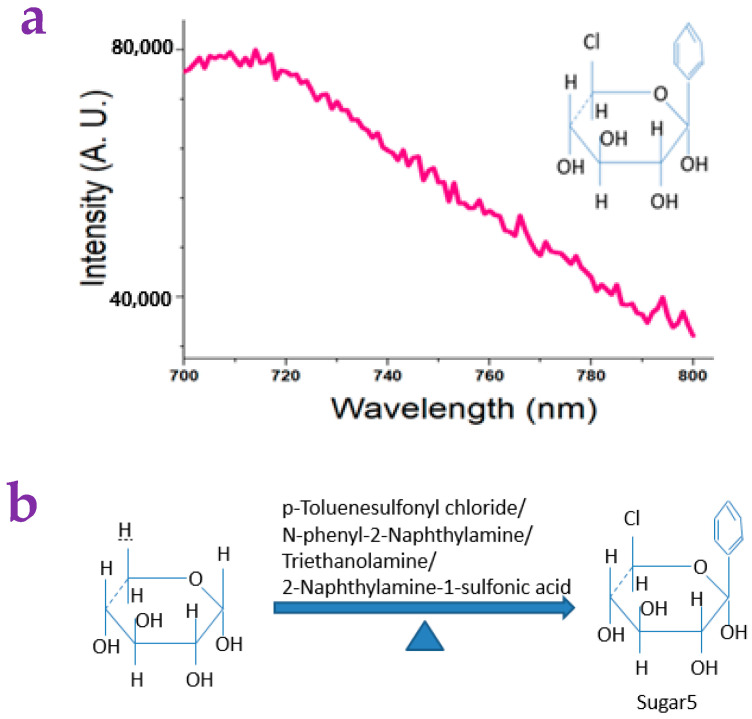
(**a**) The Sugar5 molecule showed fluorescence ranging from 700 nm to 800 nm when it was excited by a light with a wavelength of 680 nm. This was tested using a commercial fluorescence spectrometer. (**b**) The synthesis route of Sugar5.

In order to make an effective paper substrate for detecting small molecules, we used several molecules that were either synthesized or commercially available to modify the paper surface. We found that a sugar-like molecule that we synthesized could meet this requirement. A detailed synthesis of the sugar-like molecule can be found in the end of this article. Its molecular formula is shown in the inset of Figure 4a. We called this sugar-like molecule Sugar5. The synthesis route of Sugar5 is shown in Figure 4b. Sugar5 showed interesting fluorescence ranging from 700 nm to 800 nm when it was excited by a light with the wavelength of 680 nm. This may reflect its possible application in near-infrared imaging of bio-tissue.

**Figure 5:**
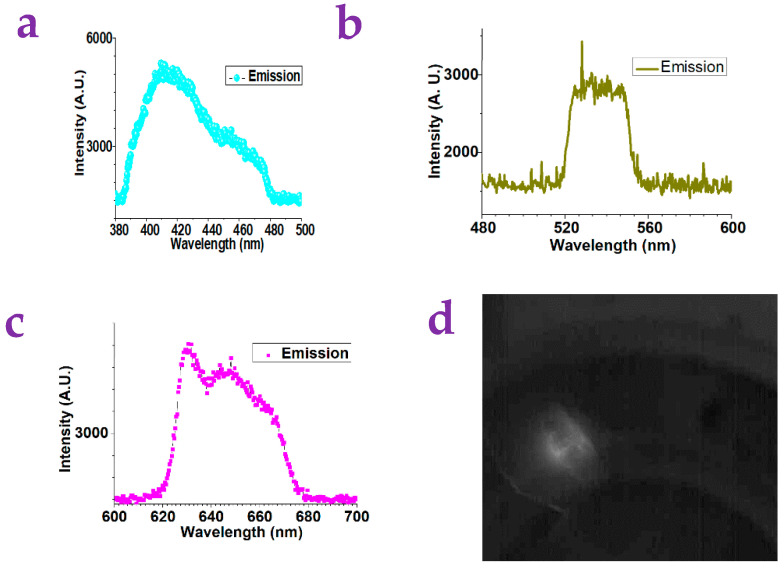
(**a**) The Sugar5 molecule showed weak emission of blue light when it was excited by a UV laser. (**b**) The Sugar5 molecule showed weak emission of green light when it was excited by a blue-light source. (**c**) The Sugar5 molecule showed red emission when it was excited by a green light. (**d**) A corn bug was injected with the Sugar5 molecule in water solution. It can be observed from a fluorescence microscope when it was irradiated with a green light.

As shown in Figure 5a–c, the Sugar5 showed blue, green, and red emission when it was excited by the UV light, the blue light, and the green light. We injected the Sugar5 molecule in water solution into a corn bug. We irradiated the corn bug with a green-light laser and used a CCD camera to observe the body of the corn bug with the red emission. Here, the green-light irradiation has been cut off by a red-light filter (600–700 nm). What we can see from the camera was actually the red fluorescence. This showed the potential of the Sugar5 on the bio-tissue imaging.

**Figure 6:**
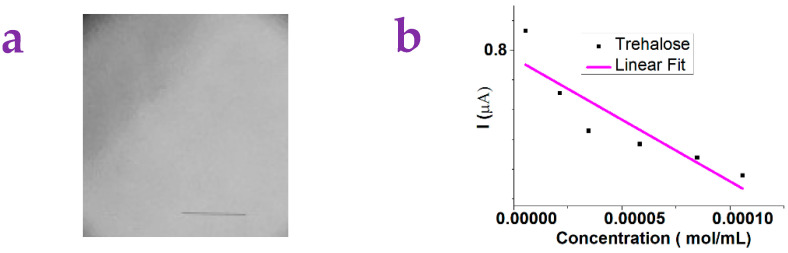
(**a**) The optical image of the paper substrates modified by Sugar5 (we called these substrates Sugar5-paper). The scale bar is 1 mm. (**b**) The photocurrent of Sugar5-paper is dependent on the concentration of the D-Trehalose. The pink curve showed a linear fit.

Sugar5 was used to modify a printing paper. The acquired paper, which we called Sugar5-paper, was used as a platform for detecting the molecules. Here, the measurement diagram is similar to the platform made by the gel. The target molecule with a certain concentration was dropped onto the surface of the Sugar5-paper. The red light was irradiated on the surface of the Sugar5-paper. The ampere meter was used to measure the photocurrent. Figure 6a showed the optical image of the Sugar5-paper surface.

Figure 6b showed that the detection of the D-Trehalose was possible given that the photocurrent showed a linear trend against the concentration of the D-Trehalose. A linear fit was conducted to derive the sensitivity, whose value is found to be −5542.4. Comparatively, no linear trend can be found for the other molecules including the Cinnamaldehyde, the Cyclohexyldiphenylphosphine, the 2, 6-Dichlorobenzaldehyde, and the Ethyl caprate, which means that the detection for these molecules is not possible (see Figure S6).

Our device was capable of detecting several types of small molecules, including amines, sugars, and organic acids. It is clear that this platform provided a new idea for the detection of small molecules.

In our device, the red-light source system consists of a red-light filter with a white-light source from a cell phone. An ampere meter measures the photocurrent. It can be seen that the red-light filter and the ampere meter can be purchased on Amazon. This allows the device to be used in remote areas.

The key part of the first device we made is to use xanthan gum modified by the metal ions as the gel substrate for detecting the small molecules. A good linear fit can be seen in the data. The key step for making the second device is to synthesize the Sugar5 molecules, which were used to modify the surface of the paper sheet.

The gels and the paper used in this work are sustainable and environmentally friendly materials. The small molecules we detected are useful for the diagnosis and prognosis of some diseases. Therefore, the device can be potentially used in biomedical engineering.

The gel and the paper materials are usually three-dimensional structures composed of polymer networks, which have characteristics of high water absorption, reversibility, biocompatibility, and mechanical property regulation. The gel and the paper materials contain a number of active sites and conductive channels that can interact with photons. This initiates a series of light-induced chemical reactions that lead to the generation of photocurrents. These properties contribute to the stabilization of photoactive species, resulting in a stable photocurrent under light irradiation.

Under the action of light, the photosensitive molecules or photo-responsive groups in the substrate will be excited by photon energy to generate electronic charge pairs. Then, in the internal structure of the substrate, the generated charges may be separated due to the electron conduction properties of the material, that is, the electrons conduct the electrical channel migration to form a negative charge region, and the positive charges migrate to other directions to form a positive charge region. The separated electrons and positive charges move along the conductive channels inside the substrate to form a charge transport, which in turn creates a potential difference. These charge transport paths are often determined by the conductive properties of the substrate materials, e.g., gels doped with conductive metal compounds may have better charge transport properties. Photocurrents are formed when the separated electrons and positive charges are transported inside the material and eventually flow through external circuits.

Gel and paper materials often have abundant pore structures and surface functional groups that can specifically recognize and bind the target of the small molecules. The small molecules may directly or indirectly affect the photocurrent of substrate materials by changing their specific chemical and photo-physical properties with respect to the change of the charge state, photosensitivity, or optical properties of the gel and paper materials. When the target molecule binds specifically to the recognition element on the substrate, it may trigger a change in the substrate structure, which may lead to a change in the charge distribution inside the substrate, affecting the separation and transport of charges, changing the electric field strength, and ultimately affecting the magnitude of the photocurrent.

The small molecules have the potential to affect photocurrent generation and performance by influencing noncovalent bonds. These noncovalent bonds typically include hydrogen bonds, van der Waals forces, π–π interactions, etc., which can affect the structure, optical properties, and electrical properties of the material, thereby affecting the generation and transmission of photocurrents.

Some small molecules may be photosensitive in their own right. When the small molecules in the substrate materials are irradiated with light of a specific wavelength, they absorb light energy, causing electrons to jump from valence to conduction bands into an excited state. These excited electrons and the positive charges left behind are then separated by an electric field, forming a freely moving charge carrier. This light-induced chemical reaction or electron transfer may alter the charge state of the gel or the paper, thus affecting the generation of the photocurrents. There are some small molecules that are photocatalytic active, which can catalyze related chemical reactions under light, which then lead to the production of new chemicals or active intermediates on the surface of the gel or the paper, which then have an impact on the photocurrent.

It can be seen that there are many reasons for the generation of photocurrents in photoelectric sensors based on the gel and the paper materials, involving the interaction between molecular recognition and binding, the generation of photo-living substances, the surface-enhancement effect, and the photo-charge separation effect. The combination of these mechanisms makes the gel and the paper important functional materials in the field of photoelectric sensors and has a wide range of application prospects.

The electro-polarization parameter is an important physical quantity to describe the polarization effect of a material under the action of an external electric field. The polarizability represents the degree to which the material responds to the external applied electric field. The polarization density describes the charge distribution inside the material, that is, the relative displacement of the positive and negative charges under the action of the external electric field. The electrical displacement represents the relationship between the electric field and the charge density distribution in the material, including the electrical displacement caused by the free charge and the electric displacement caused by the polarized charge. Together, these parameters describe the electrical properties and response behavior of materials, which are of great significance for understanding the behavior of materials under electric fields.

The electro-polarization parameters in the gel and paper materials are affected by a variety of factors. The structure and composition of the gel and the paper determine the arrangement of the molecules and particles and the responsiveness to the external electric field; the external electric field strength will directly affect the internal charge distribution and polarization degree, the temperature increase will weaken the polarization effect and lead to the decrease of the polarization parameter, the concentration of added small molecules will also affect the polarization characteristics, and the applied mechanical stress will also change the arrangement of the molecules or the particles and thus change the polarization effect.

The change of the polarization effect of the material will further affect the change of the built-in electric field of the gel and the paper. This polarization effect mainly includes two mechanisms: orientation polarization and displacement polarization. Orientation polarization refers to the fact that the molecules or the particles with a fixed direction in the gel or paper tend to be oriented in the direction of the electric field under the action of an external electric field, forming an orderly arrangement, thus generating an internal electric field. Displacement polarization refers to the influence of the electron cloud in the gel and the paper by an external electric field, resulting in an inhomogeneity of local charge distribution inside the gel and the paper, and then forming an internal electric field. These two polarization effects interact to determine the strength and direction of the built-in electric field of the gel and the paper.

The addition of small molecules to the gel or the paper material may alter the local charge distribution in the gel and the paper, which in turn alters the polarization effect of the gel and the paper. In addition, the presence of the small molecules may alter the structure and morphology of the gel and the paper, which in turn affects the arrangement of molecules or particles and the internal polarization characteristics of the gel and the paper. In addition, the small molecules may modulate the concentration or distribution of fillers in the gel and the paper, directly affecting the intensity and direction of the built-in electric field, resulting in a sometimes large or small photocurrent.

**Figure 7:**
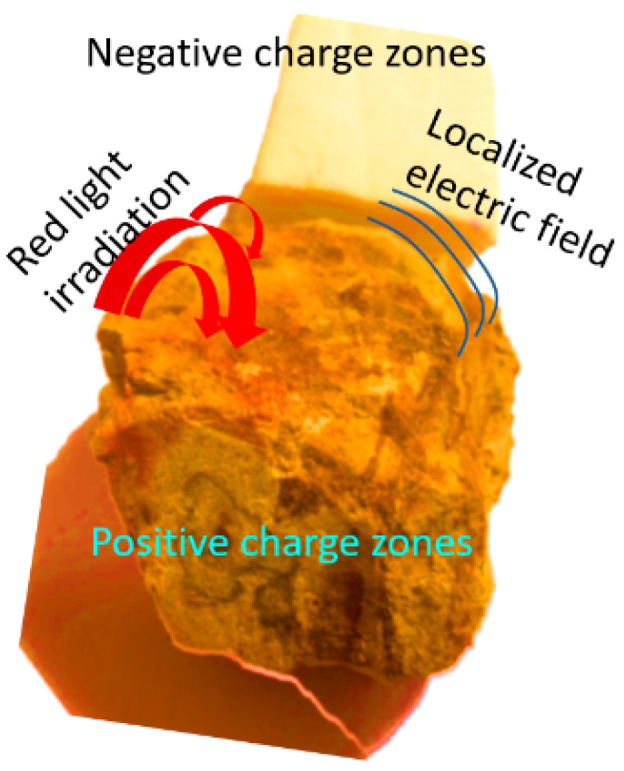
The mechanism of detecting the small molecules using our devices.

Under the conditions of the red-light radiation, negative charges and positive charges may be formed on the surface of the gel and the paper due to the presence of defects. The negative charges and the positive charges are clustered separately to form their respective regions (as shown in Figure 7). These regions form polarization parameters, respectively, because the polarization parameters are proportional to the magnitude of the current. As a result, multiple photocurrents can be generated. The superposition of these photocurrents forms the total photocurrent, which is observed by the instrument. The dropwise addition of small molecule solvents exacerbates the presence of surface defects, thus altering the photocurrent accordingly. Total photocurrent I = I_1_ − I_2_ − I_3_. Here, I_1_ is the photocurrent corresponding to the gel or the paper, I_2_ is the photocurrent introduced by metal ions, and I_3_ is the photocurrent introduced by the small molecules. The change of the value of (I_2_ + I_3_) results in an increase or decrease in I.

Previous studies of the photocurrent with respect to the concentration of small molecules are all involved with an electrochemical station, which costs around USD 20,000. There were no sensing strategies showing that an ampere meter was used. To the best of our knowledge, this is the first successful investigation of sensing small molecules directly using the simple method of photocurrent measurement. Much can be investigated in future works to further explore the in vivo sensing of small molecules and proteins. The small molecules have been considered to be important in research and development, given that they have shown rich chemical, biological, or physiological properties [31,32]. Our future endeavors would be focused on combining the advanced imaging technique, Lab-on-a-chip, and nanomaterials [33,34,35,36,37,38,39,40,41,42,43,44,45,46,47,48] to achieve high sensitivity for the detection of small molecules. The direction of our future work would be associated with a comparative analysis of the devices’ performance relative to existing methods, including the enzyme-linked immunosorbent assay [49,50], aptamer-based CRISPR-Cas powered diagnostics [51], the time-resolved fluorescence resonance energy transfer (TR-FRET) assay [52], enzyme-linked DNA displacement (ELIDIS) assay [53], and colorimetric detection [54]. Given that the xanthan Gum and the Sugar5 showed vivid fluorescence, they can be used as the substrates of fluorescence detection on the small molecules, which could also provide a comparative study of our devices’ performance. Another future direction could be conducting tests in complex biological samples (e.g., blood serum, urine), which would help clarify their selectivity and reliability in practical settings.

## 3. Conclusions

Inspired by the POC detection strategy, this work constructed two devices based on either xanthan gum or a paper sheet to detect several small molecules, including the N-phenyl-2-naphthylamine, the L-Phenylalanine, the L-(+)-arabinose, the D-(+)-Mannose, the L-Methionine, and the Trehalose molecules. Linear response can be found in the curve of the photocurrent against the concentration of the small molecules. The small molecules we detected can be used for the diagnosis and prognosis of some diseases. This work provides a novel method for the detection of small molecules in medicine and biology. Our future endeavors would focus on optimizing the detection accuracy and exploring possible equipment integration.

## 4. Materials and Methods

### 4.1. Materials

Potassium carbonate and Calcium chloride were purchased from Xilong Chemical Co., Ltd. (Shantou, China). Manganese chloride was purchased from Shanghai Yuanye Bio-Technology Co., Ltd. (Shanghai China). Zinc acetate anhydrous was purchased from Shanghai Maclin Bio-Technology Co., Ltd. (Shanghai China). Xanthan gum, Erbium (III) chloride hexahydrate, Samarium (III) chloride hexahydrate, Ferric (III) chloride hexahydrate, Cesium carbonate, Holmium (III) chloride hexahydrate, Lithium chloride, Neodymium (III) chloride hexahydrate, Ytterbium (III) chloride hexahydrate, Cerium (III) chloride heptahydrate, Europium (III) chloride hexahydrate, Chromium (III) nitrate nonahydrate, Yttrium (III) chloride hexahydrate, L-Phenylalanine, N-Phenyl-2-naphthylamine, L-Methionine, L-(+)-Arabinose, D(+)-Trehalose dehydrate, 2,6-Dichlorobenzaldehyde, Ethyl caprate, Cinnamaldehyde, Cyclohexyldiphenylphosphine, and D-(+)- Mannose were purchased from Aladdin Biochemical Technology Co., Ltd., Shanghai, China. Copper (II) nitrate hydrate was purchased from Beijing Bairuiwei Technology Co., Ltd. (Beijing China). Sodium carbonate was purchased from Alpha Aesar Chemicals Co., Ltd. (Shanghai, China). N, N-Dimethylformamide (DMF) was purchased from Sinopharm Chemical Reagent Co., Ltd. (Shanghai, China).

### 4.2. Preparation of Gel Substrates

First, xanthan gum (8 g) was added to ultrapure water (300 mL). After stirring for 30 min, the xanthan gum gel was obtained. Next, gel materials with various photoelectric responses were prepared by adding different metal ions to the gel materials. The metal reagent (0.02 g) in Table 1 was taken and added to the ultrapure water (5 mL). When mixed with the xanthan gel, the gel materials containing different metal ions were obtained.

The xanthan gum was modified by potassium ions, which is denoted as gel-K in the following discussion. We also modified the xanthan gum using other metal ions, including calcium, manganese, zinc, etc., which are referred to as gel-Ca, gel-Mn, and gel-Zn, respectively. These gels hold a similar denotation to that of the gel-K.

### 4.3. Preparation of the Solution of the Small Molecules for Detection

The N-Phenyl-2-naphthylamine was dissolved in DMF to achieve several concentration values of 8.6 × 10^−5^ mol/mL, 3.4 × 10^−4^ mol/mL, 5.6 × 10^−4^ mol/mL, 9.5 × 10^−4^ mol/L, 1.4 × 10^−3^ mol/mL, and 1.7 × 10^−3^ mol/mL, respectively. The L-Arabinose were dissolved in ultrapure water to achieve several concentration values of 1.3 × 10^−5^ mol/mL, 5.3 × 10^−5^ mol/mL, 8.7 × 10^−5^ mol/mL, 1.5 × 10^−4^ mol/mL, 2.1 × 10^−4^ mol/mL, and 2.7 × 10^−4^ mol/mL. The D-Mannose was dissolved in ultrapure water to achieve several concentration values of 1.1 × 10^−4^ mol/mL, 4.4 × 10^−4^ mol/mL, 7.2 × 10^−4^ mol/mL, 1.2 × 10^−3^ mol/mL, 1.8 × 10^−3^ mol/mL, and 2.2 × 10^−3^ mol/mL, respectively. The L-Phenylalanine was dissolved in ultrapure water to obtain several concentration values of 1.2 × 10^−5^ mol/mL, 4.8 × 10^−5^ mol/mL, 7.9 × 10^−5^ mol/mL, 1.3 × 10^−4^ mol/L, 1.9 × 10^−4^ mol/mL, and 2.4 × 10^−4^ mol/mL, respectively. L-Methionine was dissolved in ultrapure water to obtain several concentration values of 1.3 × 10^−5^ mol/mL, 5.4 × 10^−5^ mol/mL, 8.7 × 10^−5^ mol/mL, 1.5 × 10^−4^ mol/mL, 2.1 × 10^−4^ mol/mL, and 2.7 × 10^−4^ mol/mL, respectively. A hotplate associate with a temperature of 80 °C was used to heat the sample when preparing the solution of the L-Phenylalanine and the L-Methionine. The D-(+)- Trehalose-dihydrate was dissolved in ultrapure water to obtain several concentration values of 5.3 × 10^−6^ mol/mL, 2.1 × 10^−5^ mol/mL, 3.4 × 10^−5^ mol/mL, 5.8 × 10^−5^ mol/L, 8.5 × 10^−5^ mol/mL, and 1.1 × 10^−4^ mol/mL, respectively. The 2,6-Dichlorobenzaldehyde was dissolved in DMSO to gain several concentration values of 1.3 × 10^−6^ mol/mL, 5.0 × 10^−5^ mol/mL, 8.2 × 10^−5^ mol/mL, 1.4 × 10^−4^ mol/L, 2.0 × 10^−4^ mol/mL, and 2.5 × 10^−4^ mol/mL, respectively. The Ethyl caprate was dissolved in DMSO to obtain several concentration values of 1.1 × 10^−5^ mol/mL, 4.4 × 10^−5^ mol/mL, 7.1 × 10^−5^ mol/mL, 1.2 × 10^−4^ mol/L, 1.8 × 10^−4^ mol/mL, and 2.2 × 10^−4^ mol/mL, respectively. The Cinnamaldehyde was dissolved in DMSO to acquire several concentration values of 1.7 × 10^−5^ mol/mL, 6.7 × 10^−5^ mol/mL, 1.1 × 10^−4^ mol/mL, 1.8 × 10^−4^ mol/L, 2.7 × 10^−4^ mol/mL, and 3.3 × 10^−4^ mol/mL, respectively. The Cyclohexyldiphenylphosphine was dissolved in DMSO to obtain several concentration values of 8.2 × 10^−6^ mol/mL, 3.3 × 10^−5^ mol/mL, 5.3 × 10^−5^ mol/mL, 9.0 × 10^−5^ mol/L, 1.3 × 10^−4^ mol/mL, and 1.6 × 10^−4^ mol/mL, respectively. All the small molecule solutions with the concentrations listed above were used for our experiment.

### 4.4. Setting up an Optical Microscope

A lab-built optical microscope was used to view the surface of the sample. A white-light source was irradiating the sample surface. An objective (10×) that was attached to a moving stage was put above the sample surface. The amplified sample surface was seen from a CCD, which was put above the objective.

### 4.5. Collection of Blue Fluorescence

A UV laser (350–355 nm) was purchased from Gainlaser, Inc., Shenzhen, China. It was irradiated from the sample side. A biconvex mirror was put above the sample, which would collect fluorescence and focus the fluorescence to an optical fiber, which was connected with an optical spectroscope (Model: FX2000, Company: Shanghai Fuxiang Optical Co., Ltd., Shanghai, China). A blue-light filter (380–480 nm) was connected with the optical fiber, which would reduce the UV-light irradiation.

### 4.6. Collecting Green Fluorescence

The collection of green fluorescence is similar to that of blue fluorescence. An LED array was used to provide blue-light irradiation. A plastic optical fiber (diameter: 1 cm) was coupled with the LED array, which would transmit the blue light. The blue light was irradiated on the side of the sample. A biconvex mirror was put above the sample, which would collect the fluorescence and focus the fluorescence to the optical fiber, which was connected with the optical spectrometer. A green-light filter (520–560 nm) was connected with the optical fiber, which would reduce the blue-light irradiation.

### 4.7. Collecting Red Fluorescence

The collection of red fluorescence is similar to that of blue fluorescence. A green-light laser (532 nm) was used to provide green-light irradiation on the sample side. A bioconvex mirror was put above the sample, which would collect the fluorescence and focus the fluorescence to the optical fiber, which was connected with the optical spectrometer. A red-light filter (600–700 nm) was connected with optical fiber, which would reduce the green-light excitation.

### 4.8. Imaging of the Corn Bug with Red Fluorescence

The corn bug was injected with the Sugar5 in the water solution. The green-light laser (532 nm) was used to irradiate the corn bug. The red-light filter (600–700 nm) was put above the corn bug to reduce the green-light irradiation. A bioconvex mirror was put above the red-light filter and the image was focused with red fluorescence on a camera.

### 4.9. Fabrication of the Device Based on the Gels for Detecting the Small Molecules

The xanthan gum (8 g) was dissolved in ultrapure water (300 mL) to obtain gel1. The metal compound (0.02 g) was dissolved in ultrapure water (5 mL) to obtain Metal1. gel1 and Metal1 were mixed to acquire gel2. gel2 (0.5 g) was coated on a plastic surface to obtain the final sample with a dimension of 3 cm × 2 cm. Copper wires were inserted into the two ends of the gels. The white light from the cell phone passed through the red-light filter, which generated red light. The cell phone and the filter were fixed in the same position during the entire experiment in order to generate the same light irradiation, whose power was approximately 100 mW. When the gel substrate (0.5 g, 3 cm × 2 cm) was exposed to the red light, the photocurrent was generated and detected by the ampere meter. When the small molecules with different concentrations were added to the gel substrate, the photocurrent could be changed.

### 4.10. Synthesis of Sugar5

A hotplate whose temperature was set to 280 °C was used for providing heating during the preparation. A beaker (50 mL) was used to hold the samples. A Teflon rod was held in hand to stir the samples constantly during preparation. The p-Toluenesulfonyl chloride (0.1 g) was dissolved in ultrapure water (5 mL). The D-(+)-Xylose (1 g), the ethanol (95%, 15 mL), the Triethanolamine (5.2 g), the 2-Naphthylamine-1-sulfonic acid (0.6 g), and the Dimethylamine hydrochloride (1.2 g) were added subsequently. When all the samples in the beaker were dissolved, the D-(+)-Xylose (2 g) was added again. After heating and stirring for 3 min, the N-phenyl-2-Naphthylamine (1.6 g), the ethanol (95%, 15 mL), the ultrapure water (10 mL), and the Dimethylamine hydrochloride (0.8 g) were added afterward. Then, the D-(+)-Xylose (0.6 g) was added one more time, and the Triethanolamine (6.8 g) was added in a second time. The acquired gray solution was heated and stirred for 0.5 h to obtain the final black sample. Extraction of the sample was performed using ethyl acetate and water (Volume ratio = 1:1). Thin layer chromatography (TLC) was performed to confirm the purification of the sample. The purified sample was characterized by the 13C NMR and HNMR, which we denoted as Sugar5. The molecular structure of Sugar5 was derived based on the data of the 13C NMR and HNMR. We obtained the NMR values as follows: 1H NMR (400 MHz, Chloroform-d) δ 3.57 (s, 1H), 2.71–1.74 (m, 1H), 1.32 (s, 1H), 0.93 (d, J = 20.0 Hz, 0H), 0.11 (d, J = 28.3 Hz, 1H). 13C NMR (101 MHz, Chloroform-d) δ 142.70, 140.60, 134.57, 129.52, 129.29, 127.70, 126.60, 123.69, 121.76, 120.11, 118.50, 111.99, 77.29, 77.03, 51.00, 1.10.

### 4.11. Fabrication of the Device Based on the Sugar5-Paper for Detecting the Small Molecules

An ordinary A4 printer paper was cut into many sheets. The dimension of every paper sheet was 1 cm × 2 cm. The Sugar5 (0.04 g) was dissolved in the ethanol (95%, 10 g). The acquired Sugar5 solution (150 μL) was dropped on the paper sheet by a pipette five times. Waiting for 3 min, this modified the paper sheet to obtain the Sugar5-paper, which would be used for further detecting of the small molecules. Here, we also measured the photocurrent against the concentration of the small molecules. We used the same diagram in the photocurrent measurement of the gels substrate for detecting the small molecules. When the small molecules with different concentrations were dropped to the Sugar5-paper substrate, the photocurrent could be changed. We recorded these values of the photocurrent with respect to the concentrations of the molecules.

## Figures and Tables

**Table 1:** Dependence on concentration of N-Phenyl-2-naphthylamine with respect to the photocurrent of gels.

Gels	Dependence	Gels	Dependence	Gels	Dependence
gel-Cr	Nonlinear	gel-Ce	Nonlinear	gel-Li	Nonlinear
gel-Na	Nonlinear	gel-Ca	Nonlinear	gel-Er	Nonlinear
gel-Eu	Nonlinear	gel-Zn	Nonlinear	gel-Cs	Nonlinear
gel-Y	Nonlinear	gel-Fe	Linear	gel-Yb	Nonlinear
gel-Sm	Nonlinear	gel-Ho	Nonlinear	gel-Mn	Linear
gel-Cu	Nonlinear	gel-Nd	Nonlinear	gel-K	Nonlinear

**Table 2:** Dependence on concentration of L-Arabinose with respect to the photocurrent of gels.

Gels	Dependence	Gels	Dependence	Gels	Dependence
gel-Cr	Nonlinear	gel-Ce	Nonlinear	gel-Li	Nonlinear
gel-Na	Nonlinear	gel-Ca	Nonlinear	gel-Er	Nonlinear
gel-Eu	Nonlinear	gel-Zn	Nonlinear	gel-Cs	Nonlinear
gel-Y	Nonlinear	gel-Fe	Linear	gel-Yb	Nonlinear
gel-Sm	Nonlinear	gel-Ho	Linear	gel-Mn	Nonlinear
gel-Cu	Nonlinear	gel-Nd	Nonlinear	gel-K	Nonlinear

**Table 3:** Dependence on concentration of D-Mannose with respect to the photocurrent of gels.

Gels	Dependence	Gels	Dependence	Gels	Dependence
gel-Cr	Linear	gel-Ce	Nonlinear	gel-Li	Nonlinear
gel-Na	Nonlinear	gel-Ca	Nonlinear	gel-Er	Nonlinear
gel-Eu	Nonlinear	gel-Zn	Nonlinear	gel-Cs	Nonlinear
gel-Y	Nonlinear	gel-Fe	Nonlinear	gel-Yb	Linear
gel-Sm	Nonlinear	gel-Ho	Nonlinear	gel-Mn	Linear
gel-Cu	Nonlinear	gel-Nd	Nonlinear	gel-K	Nonlinear

**Table 4:** Dependence on concentration of L-Phenylalanine with respect to the photocurrent of gels.

Gels	Dependence	Gels	Dependence	Gels	Dependence
gel-Cr	Nonlinear	gel-Ce	Linear	gel-Li	Nonlinear
gel-Na	Nonlinear	gel-Ca	Nonlinear	gel-Er	Nonlinear
gel-Eu	Nonlinear	gel-Zn	Nonlinear	gel-Cs	Nonlinear
gel-Y	Nonlinear	gel-Fe	Linear	gel-Yb	Linear
gel-Sm	Linear	gel-Ho	Nonlinear	gel-Mn	Nonlinear
gel-Cu	Nonlinear	gel-Nd	Nonlinear	gel-K	Nonlinear

**Table 5:** Dependence on concentration of L-Methionine with respect to the photocurrent of gels.

Gels	Dependence	Gels	Dependence	Gels	Dependence
gel-Cr	Linear	gel-Ce	Nonlinear	gel-Li	Nonlinear
gel-Na	Nonlinear	gel-Ca	Nonlinear	gel-Er	Nonlinear
gel-Eu	Nonlinear	gel-Zn	Nonlinear	gel-Cs	Nonlinear
gel-Y	Nonlinear	gel-Fe	Nonlinear	gel-Yb	Nonlinear
gel-Sm	Linear	gel-Ho	Nonlinear	gel-Mn	Nonlinear
gel-Cu	Nonlinear	gel-Nd	Nonlinear	gel-K	Nonlinear

## Data Availability

Data can be provided upon reasonable request.

## Supplementary Materials

The following supporting information can be downloaded at: https://www.mdpi.com/article/10.3390/gels10120808/s1, Figure S1: The photocurrent of various gel substrates can be depended on the concentration of N-Phenyl-2-naphthylamine; Figure S2: The photocurrent of various gels substrates can be depended on the concentration of L-Arabinose; Figure S3: The photocurrent of various gels substrates can be depended on the concentration of D-Mannose; Figure S4: The photocurrent of various gels substrates can be depended on the concentration of L-Phenylalanine; Figure S5: The photocurrent of various gels substrates can be depended on the concentration of L-Methionine; Figure S6: Several molecules were tested for the photocurrent of Sugar5-paper; Figure S7: The schematic diagram of the device based on the xanthan gum for detecting the small molecules; Figure S8: The prepared xanthan gum (without metal modification); Figure S9: Sketch of the device of Xanthan gum; Figure S10: Sketch of the device of Sugar5/paper.
