# Supplementary material for: Detecting N-Phenyl-2-Naphthylamine, L-Arabinose, D-Mannose, L-Phenylalanine, L-Methionine, and D-Trehalose via Photocurrent Measurement
Source: Gels. 2024 Dec 9;10(12):808. doi: 10.3390/gels10120808 (PMC11675501; doi:10.3390/gels10120808)
Supplement: Supplementary file 1 [file gels-10-00808-s001.zip › gels-3272987-supplementary.pdf]

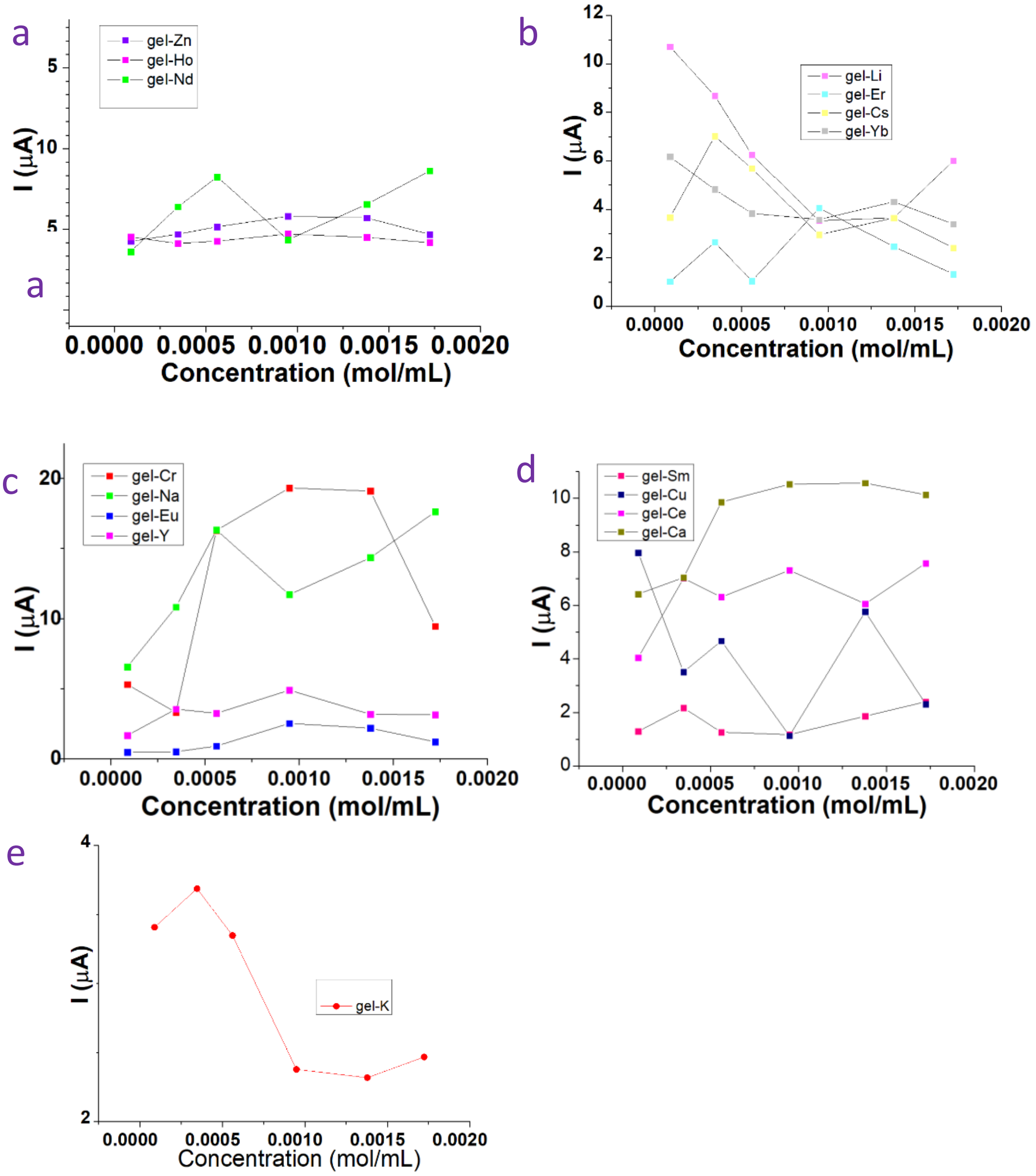

Figure S1. The photocurrent of various gel substrates can be depended on the concentration of N-Phenyl-2-naphthylamine. (a) gel-Zn, gel-Ho, and gel-Nd. (b) gel-Li, gel-Er, gel-Cs, and gel-Yb. (c) gel-Cr, gel-Na, gel-Eu, and gel-Y. (d) gel-Sm, gel-Cu, gel-Ce, and gel-Ca. (e) gel-K.

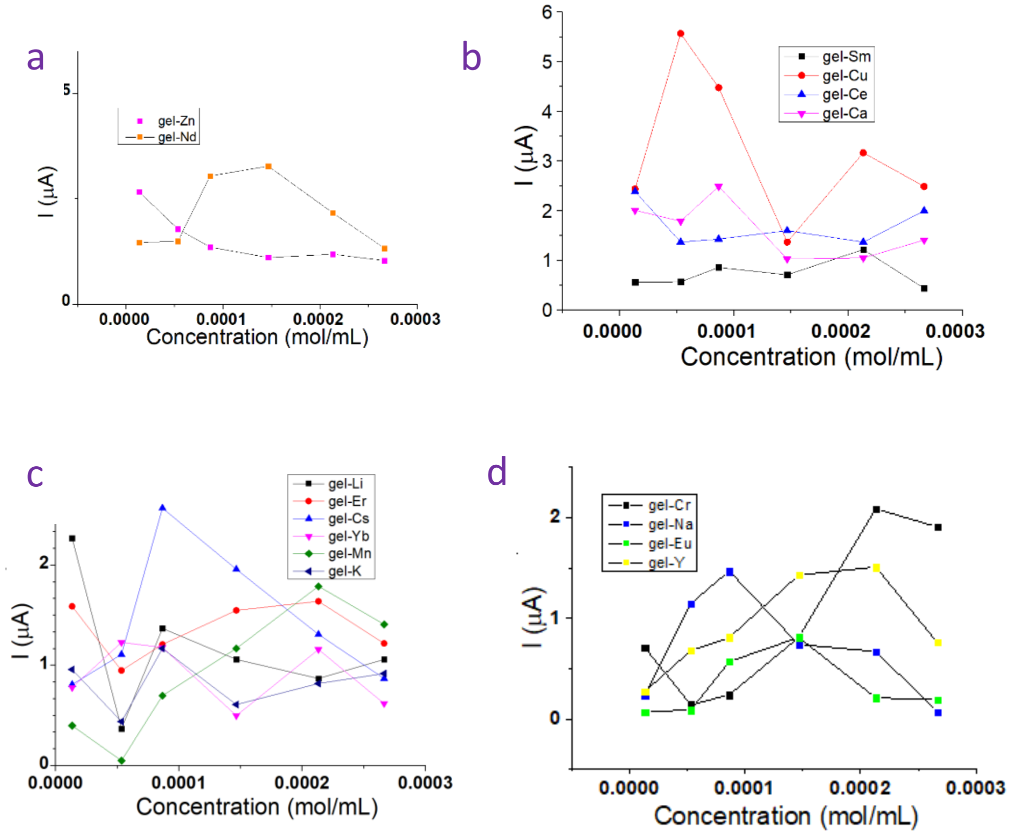

Figure S2. The photocurrent of various gels substrates can be depended on the concentration of L-Arabinose. (a) gel-Zn, gel-Nd. (b)gel-Sm, gel-Cu, gel-Ce,gel-Ca. (c) gel-Li, gel-Er, gel-Cs, gel-Yb, gel-Mn,gel-K. (d) gel-Cr, gel-Na, gel-Eu, gel-Y.

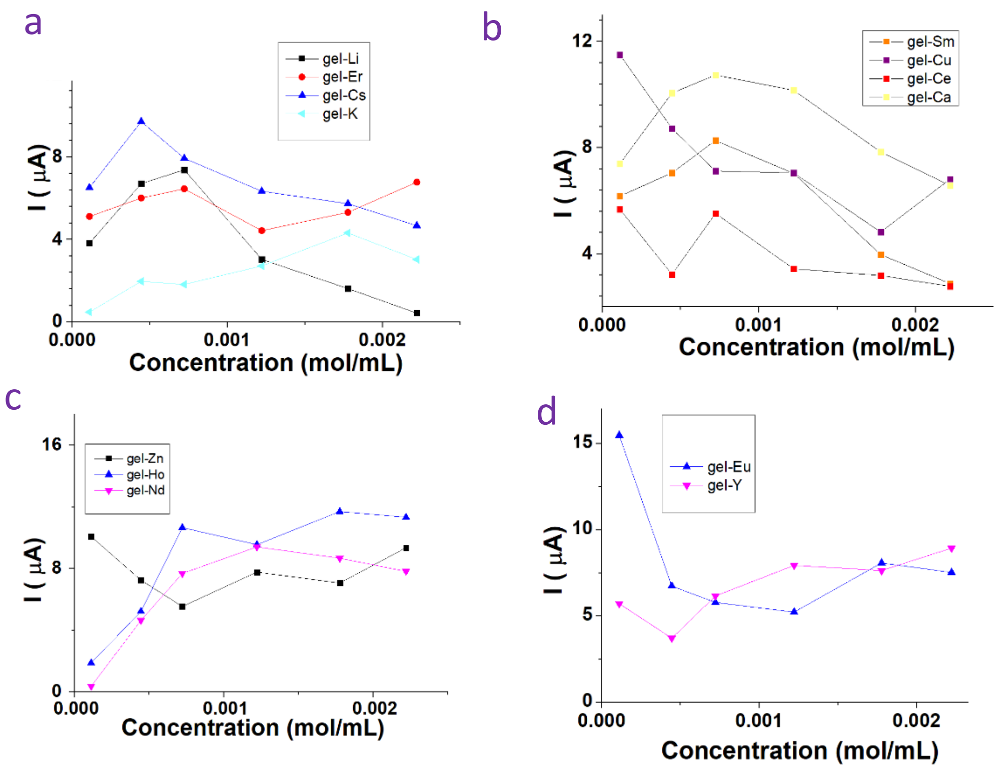

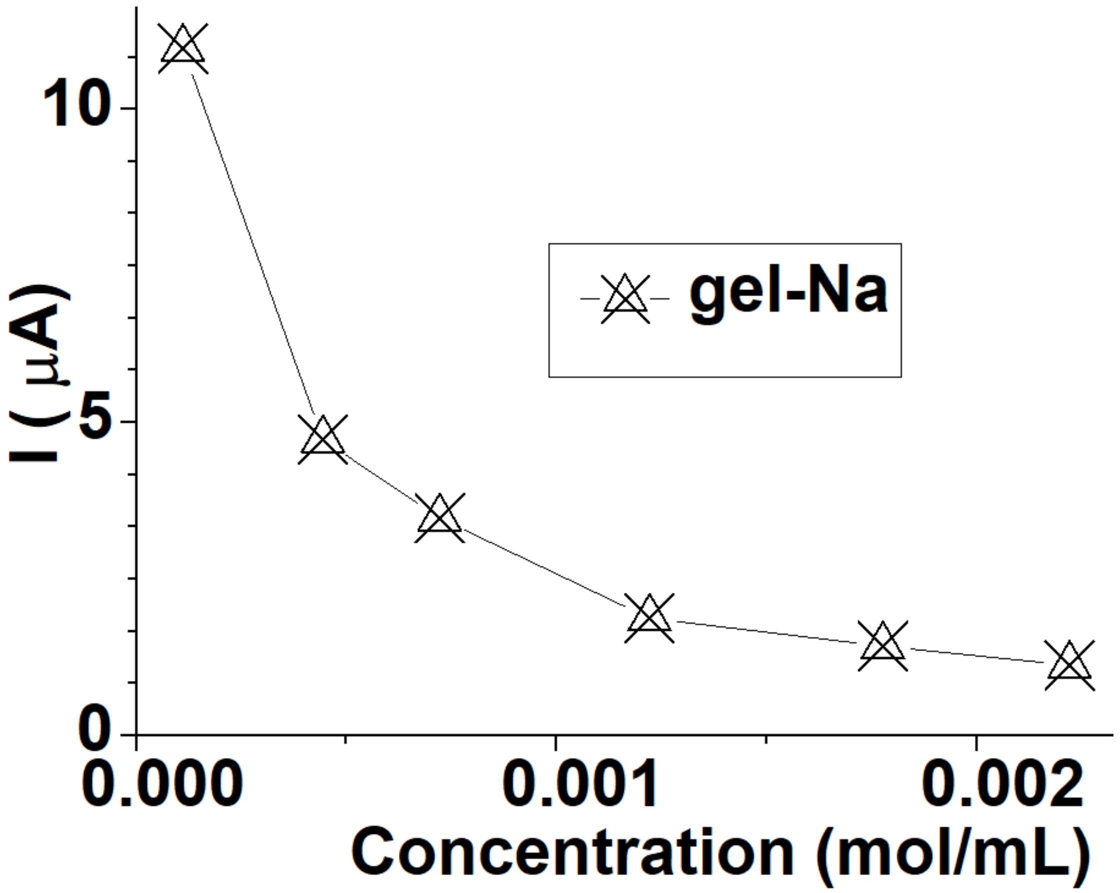

Figure S3. The photocurrent of various gels substrates can be depended on the concentration of D-Mannose. (a) gel-Li, gel-Er, gel-Cs, gel-K. (b) gel-Sm, gel-Cu, gel-Ce, gel-Ca. (c) gel-Zn, gel-Ho, gel-Nd. (d) gel-Eu, .

(e) gel-Na.

a

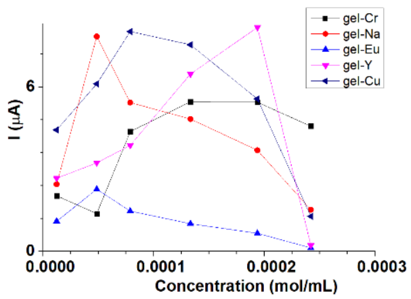

b

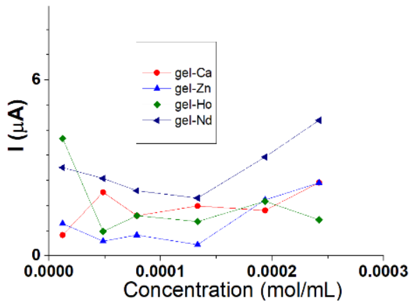

c

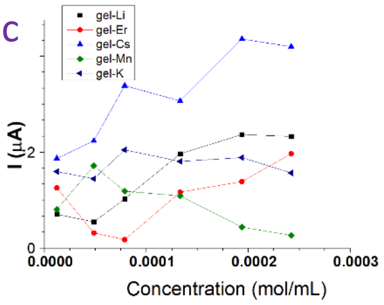

Figure S4. The photocurrent of various gels substrates can be depended on the concentration of L-Phenylalanine. (a) gel-Cr, gel-Na, gel-Eu, gel-Y, gel-Cu. (b) gel-Ca,gel-Zn, gel-Ho, gel-Nd. (c) gel-Li, gel-Er, gel-Cs, gel-Mn, gel-K.

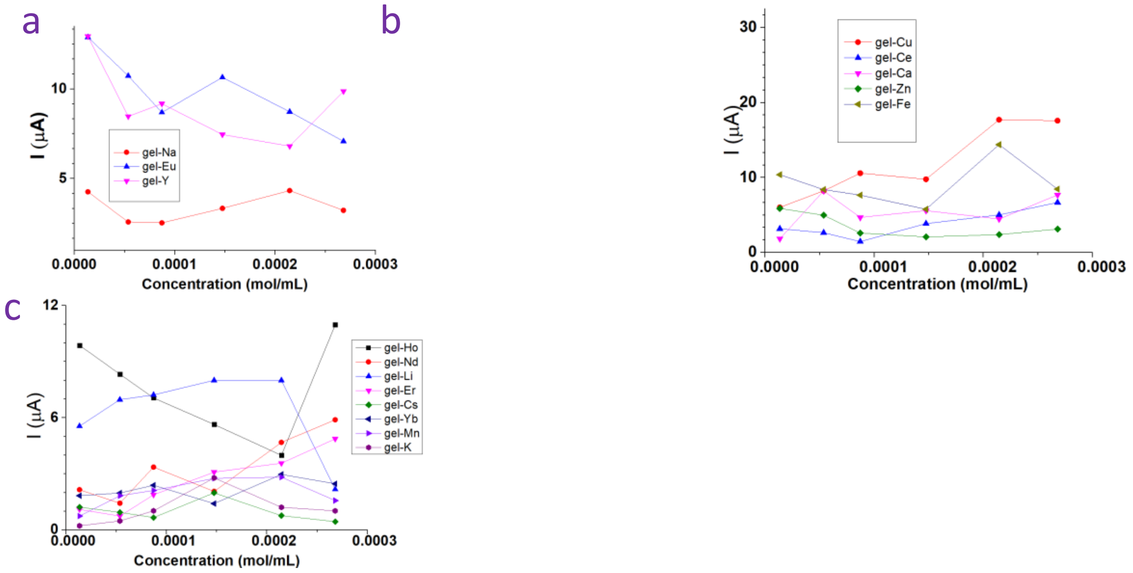

Figure S5. The photocurrent of various gels substrates can be depended on the concentration of L-Methionine.

(a) gel-Na, gel-Eu, gel-Y. (b) gel-Cu, gel-Ce, gel-Ca, gel-Zn, gel-Fe.(c) gel-Ho, gel-Nd, gel-Li, gel-Er, gel-Cs, gel-Yb, gel-Mn, gel-K.

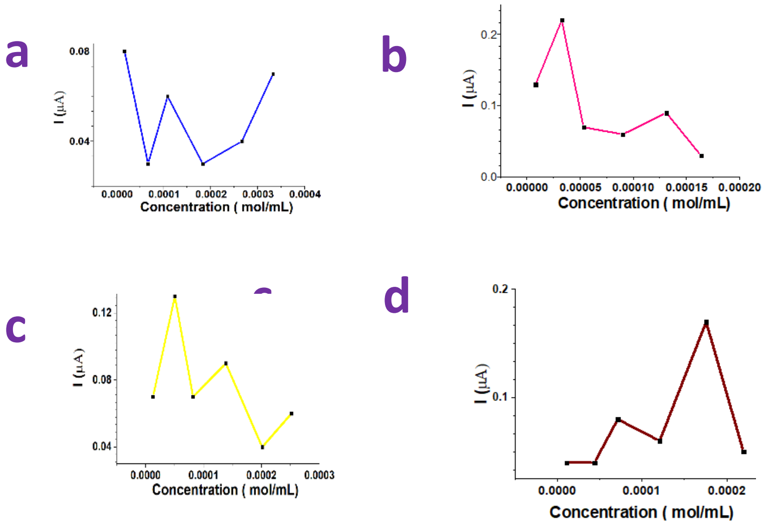

Figure S6. Several molecules were tested for the photocurrent of Sugar5-paper. (a) The photocurrent of Sugar5-paper is depended on the concentration of the Cinnamaldehyde. No linear trend was observed. (b) The photocurrent of Sugar5-paper is depended on the concentration of the Cyclohexyldiphenylphosphine. No linear trend was observed. (c) The photocurrent of Sugar5-paper is depended on the concentration of the 2,6-Dichlorobenzaldehyde. No linear trend was found. (d) The photocurrent of Sugar5-paper is depended on the concentration of the Ethyl caprate. No linear trend was observed.

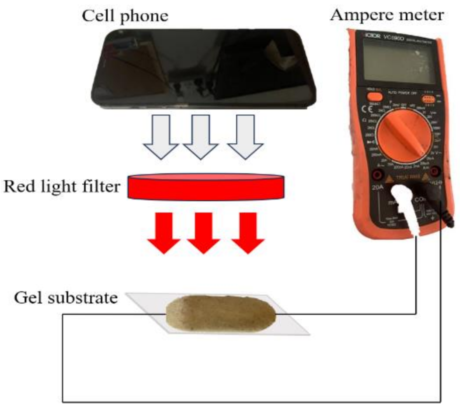

Figure S7. The schematic diagram of the device based on the xanthan gum for detecting the small molecules

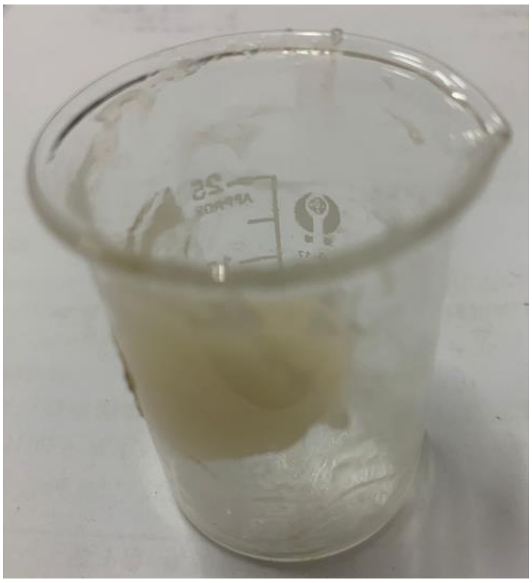

Figure S8. The prepared xanthan gum (without metal modification). This sample can attach to the wall of the beaker, which clearly showed that it is a gel not a dispersed solution.

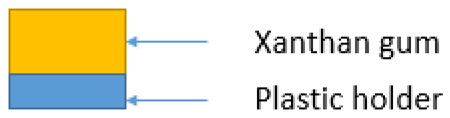

Figure S9. Sketch of the device of Xanthan gum. Here, printing paper was put in the end of the Xanthan gum, which would support the Xanthan gum.

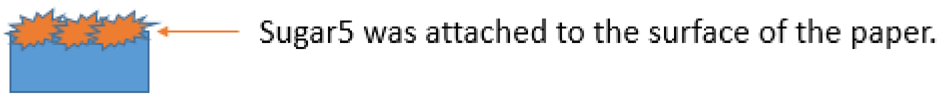

Figure S10. Sketch of the device of Sugar5/paper. Here, the paper surface was washed by the Sugar5 solution, which will make Sugar5 attached to the surface of the paper.
